# Editor Dental Register

**Published:** 1886-04

**Authors:** 


### CORRESPONDENCE.

Pasadena, Los Angeles Co., California,
March, 15th, 1886.

Editor Dental Register:

I see by one of our Pacific Coast papers that the Eexecutive Committee of the American Dental Association (to whom was delegated the power at Minneapolis of selecting a place of meeting) have selected San Francisco as the place, and the usual time, August.

While I am delighted with this selection as to place, I, (at present a dweller upon this beautiful coast,) think, all who will come here from east of the Rockies, would enjoy the beauties of this country far more if the time could be postponed to about the holiday season, for then California, especially the southern portion, which many will delight to visit, is supremely redolent with the blossoms of the orange and lemon, and the almost endless variety of semi-tropical plants and flowers, indescribably beautiful, and wonderfully comfortable in temperature. Most people East I believe have a very fallacious idea of what they please to call the rainy season of California. This is my second winter in California, and I am decidedly of the opinion that Helen Hunt Jackson was not far wrong when she said that "In California, summer is the season when it cannot rain, but sometimes does, while winter is the season when it can rain but sometimes does not." Last winter in all my travels through the State, I do not remember to have used an umbrella once, and still sufficient rain fell in nearly all portions of the State, but mostly in the night, giving hired coolies a chance to rest, and travelers a chance to sleep.

This winter has not been quite so propitious, for since the beginning of the first rain of the season, November 15th, we have had twenty days in which rain fell, some of which it rained all day or nearly so, more of which, however, it rained only a portion of the time, and still the old inhabitants here have designated it as a wet winter.

But think of it, four months (120 days), only twenty of which have been rainy, most of the others having been, in the main, bright, balmy, and cloudless, and then call the winter season of California a wet one! Why, might just as well speak of May and June in the East as a wet season, because it does often rain in those months. I write this that my Eastern brethren may know that they may safely come to California in winter without bringing their canoes with them. And while I had not as yet spent a summer in California, I know from the testimony of others that it is decidedly a dry season. August, in San Francisco particularly, is a windy, dusty, and disagreeable season, while in the winter months, when nearly all would be glad to get away from the rigors of the East, and when railroad fares are most likely to be at the minimum, you can come to California with the full assurance of having a most delightful time.

To-day I notice that first-class rates from Chicago to San Francisco are $32.50, and East bound still lower, being only $31.00 to New York. It is true there is no assurance of such low rates prevailing another winter, but this much is true, a war of rates is much more likely to occur in winter when travel can be stimulated, than in summer, when there is so little to induce travel this way.

If the above meets the eyes of the Executive Committee, as I hope it may, and they can manage to change the date as suggested, I am very sure it will meet the general approval of the members of our American Dental Association."

GEO. R. THOMAS, D.D.S.

### The American Dental Association.

